# New species of leaf-mining *Phyllonorycter* (LepidopteraGracillariidae) from Siberia feeding on *Caragana* (Fabaceae)

**DOI:** 10.3897/zookeys.835.33166

**Published:** 2019-04-04

**Authors:** Natalia Kirichenko, Paolo Triberti, Carlos Lopez-Vaamonde

**Affiliations:** 1 Sukachev Institute of Forest SB RAS, Akademgorodok 50/28, 660036, Krasnoyarsk, Russia; 2 Siberian Federal University, 79 Svobodny pr., 660041, Krasnoyarsk, Russia; 3 INRA, UR0633 Zoologie Forestière, F-45075 Orléans, France; 4 Museo Civico di Storia Naturale, Lungadige Porta Vittoria 9, I37129, Verona, Italy; 5 Institut de Recherche sur la Biologie de l’Insecte, CNRS UMR 7261, Université de Tours, UFR Sciences et Techniques, 37200 Tours, France

**Keywords:** Leaf-mining micromoths, legume, DNA barcoding, male genitalia morphology, Siberian peashrub

## Abstract

During a DNA barcoding campaign of leaf-mining Gracillariidae from the Asian part of Russia, a new species of *Phyllonorycter* Hübner, feeding on the Siberian pea shrub, *Caraganaarborescens* Lam. (Fabaceae) was discovered in Siberia. Here, this taxon is described as *Phyllonorycterivani***sp. n.** Among Fabaceae-feeding *Phyllonorycter*, so far only *P.caraganella* (Ermolaev) has been known to develop on *Caragana*. *Phyllonorycterivani* and *P.caraganella* show a large divergence in morphology (external and male genitalia) and barcode region of the mtDNA-COI gene (8.6%). They feed on different host plants species and have different ranges in Russia. We show that DNA barcode data weakly supports the Fabaceae-feeding species groups. In addition, we show that morphologically (strongly) and genetically (weakly), *P.ivani* has affinity to the *haasi* species group, a West Palearctic group with asymmetrical male genitalia.

## Introduction

Siberia represents approximately 9% of Earth’s land surface, and its vast boreal forests contain a diverse insect fauna with Lepidoptera being particularly well represented, accounting over 5000 species (Sinev 2008). Among Lepidoptera, micromoths show high species richness with some species being agricultural and forest pests and invaders (Kuznetzov 1999; Kirichenko et al. 2018a). Despite their ecological and economic importance, micromoths remain largely understudied (Sinev 2008; Lees et al. 2013; Lopez-Vaamonde et al. 2018).

In Siberia, leaf-mining micromoths and particularly the economically important family Gracillariidae have been the focus of recent studies, using DNA barcoding as a main tool to discover new species and host plant associations (Kirichenko et al. 2016, 2017, 2018b, 2018c, 2019; Akulov et al. 2018; Knyazev et al. 2018). Among Gracillariidae, the genus *Phyllonorycter* Hübner, 1822 is the most diverse, with more than 400 species described worldwide (De Prins and De Prins 2018) and over 200 species recorded from the Asian part of Russia (Baryshnikova 2008, 2016; Kirichenko et al. 2019), feeding on plants from various families (De Prins and De Prins 2018).

Legumes (Fabaceae) belonging to eight tribes (Desmodieae, Fabeae, Genisteae, Hedysareae, Loteae, Phaseoleae, Robinieae, and Trifolieae) (Roskov et al. 2019) have been known as hosts for 57 *Phyllonorycter* species that are mainly distributed in the Palearctic (51 species) and a few species found in the Nearctic (3), Afrotropics (2) and Indomalaya (1) (Suppl. material 1: Table S1). Of these 57 species, 39 (i.e., 68%) are known to be strictly monophagous, feeding on a single legume species (Suppl. material 1: Table S1).

The majority of the Palearctic Fabaceae-feeding *Phyllonorycter* (48 species, i.e., 84%) have asymmetrical male genitalia (Suppl. material 1: Table S1), with a large left valva showing a pronounced spine at apex and a narrow right valva, with almost parallel costal and ventral margins. A small group of five species has symmetrical genitalia with thin and parallel-sided valvae (Suppl. material 1: Table S1). All species with the asymmetric male genitalia, except *P.nigrescentella* (Logan, 1851), *P.insignitella* (Zeller, 1846), *P.tangerensis* (Stainton, 1872), and *P.viciae* (Kumata, 1963), and one species with symmetrical male genitalia, *P.cerasinella* (Reutti, 1853) develop on legumes belonging to the Genisteae (Laštůvka and Laštůvka 2006). This is a highly diverse tribe of the subfamily Faboideae, largely distributed in western Palearctic (Ainouche et al. 2003). An extensive study of the *Phyllonorycter* species developing on Genistae defined three groups based on morphology of male genitalia: the *haasi*, *fraxinella*, and *ulicicolella* groups, all including species with asymmetrical male genitalia (Laštůvka and Laštůvka 2006). Subsequent phylogenetic analysis reconsidered the placement of *P.phyllocytisi* (Hering, 1936), *P.eugregori* Laštůvka & Laštůvka, 2006, *P.telinella* Laštůvka & Laštůvka, 2006, and *P.nevadensis* (Walsingham, 1908) (Laštůvka et al. 2013) that do not fit in any of those species groups and thus occupy a relatively isolated position.

Among Fabaceae-feeding *Phyllonorycter*, *P.caraganella* (Ermolaev, 1986) is the only species known to feed on the legume genus *Caragana* (De Prins and De Prins 2018). This plant genus belongs to the tribe Hedysareae, a clade significantly divergent from Genisteae (LPWG et al. 2009). *Phyllonoryctercaraganella*, of which the males have symmetrical genitalia, develops on *Caraganamanshurica* Kom. and is found exclusively in the Russian Far East, in the southern part of Primorsky Krai, the region bordering with Northeast China (Ermolaev 1986; Baryshnikova 2016).

During fieldwork in Central and Eastern Siberia, we collected two *Phyllonorycter* larvae mining leaves of the Siberian pea shrub, *C.arborescens* Lam. Analysis of the DNA barcodes of those two larvae revealed a large molecular divergence with DNA barcodes of *P.caraganella*. Further sampling and rearing and detailed morphological examination of adults confirmed the existence of a new *Phyllonorycter* species feeding on *C.arborescens*. Here we provide the description of this new species, *Phyllonorycterivani* Kirichenko, Triberti & Lopez-Vaamonde sp. n. and expand the morphological description of *P.caraganella* from the Russian Far East. We also investigate whether DNA barcodes support the different Fabaceae-feeding *Phyllonorycter* species groups that have been based on the morphology of male genitalia.

## Materials and methods

### Sampling

**Figure 1.**
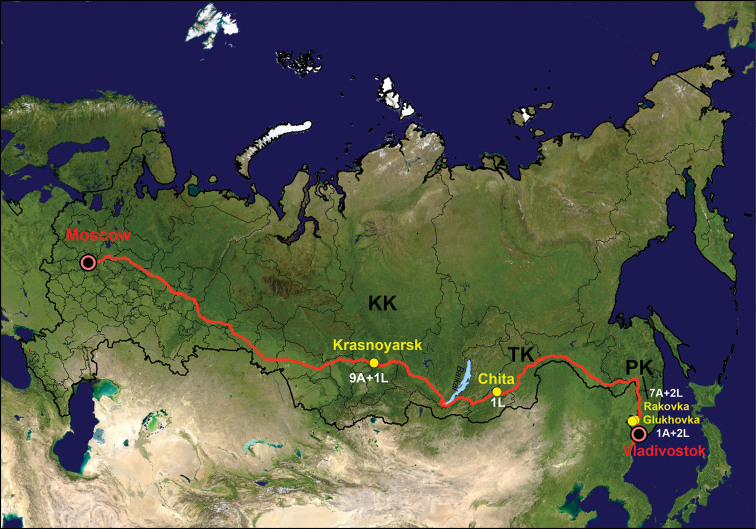
The study region in Russia. Sampling locations are indicated by yellow circles: Krasnoyarsk and Chita for *Phyllonorycterivani*, Rakovka and Glukhovka for *P.caraganella.* Number of specimens is given for each sampled location: A adults, L larva. Regions: **KK** Krasnoyarsk Krai, **TK** Transbaikal (Zabaikalsky) Krai, **PK** Primorsky Krai. The Trans-Siberian railway (the total distance of 9288.2 km between Moscow to Vladivostok) is shown by red line.

Leaves with mines of *P.ivani* were collected in Central Siberia in Krasnoyarsk Krai (in the suburb of the city Krasnoyarsk, along the Yenisei river bank) and in Eastern Siberia in Transbaikal Krai (in the city Chita, Victory park) on *C.arborescens* from July to August 2014–2016 (Fig. 1). These two locations are over 2000 km apart along Trans-Siberian railway (Fig. 1). In addition, leaf mines with *P.caraganella* were collected in the Russian Far East, in the southern part of Primorsky Krai in two neighbouring locations near by the villages Glukhovka and Rakovka on *Caraganamanshurica* in July 2016 (Fig. 1).

In total, six larvae (two *P.ivani* and four *P.caraganella*) were preserved in 96% ethanol and 17 adult moths (nine *P.ivani* and eight *P.caraganella*) were reared from mines (Fig. 1, Suppl. material 2: Table S2). To obtain adults, leaves with mines containing mature larvae were kept in the plastic boxes (200ml) at constant conditions (22 °C, 55% RH, L:D 18:6 h photoperiod) following Ohshima’s (2005) protocol. Additionally, 20 leaves with mines (some leaves with larvae in mines) were placed in the annotated herbarium collection in SIF SB RAS. In June-July 2015–2017, an extensive survey of *Caragana* growing in the botanical gardens, city plantations and forests was carried out in Siberia (Tyumen Oblast, Khanty-Mansy Autonomous Okrug, Tomsk Omsk, Novosibirsk, Kemerovo Oblasts, Altai Krai, the Republics of Tuva and Buryatia, Irkutsk Oblast) and in the Russian Far East (Amur Oblast, Sakhalin Island) to check for presence of *Phyllonorycter* mines on leaves.

### Morphology and nomenclature

We examined the morphology of 17 dried and pinned specimens belonging to *P.ivani* (nine specimens) and *P.caraganella* (eight specimens). The adults of both species were photographed with Leica digital microscope DMS1000 and the incorporated digital camera and processed using the stacking system software Leica Application Suite LAS X. Genitalia were dissected from five *P.ivani* and four *P.caraganella* moths (Suppl. material 2: Table S2) and their photographs were taken with Sony Nex3 Camera from Carl Zeiss Stemi DV4 Stereo Microscope. Leaf mines were photographed in the field and in the laboratory using a digital camera Sony Nex3. All images were edited in Adobe Photoshop CS5 Extended.

Genitalia dissection and slide mounting followed Robinson (1976). Terminology of the genitalia followed Klots (1970) and Kristensen (2003).

### Specimen depositories

**MSNV**Museo Civico di Storia Naturale, Verona, Italy.

**SIF SB RAS** Sukachev Institute of Forest, Siberian Branch of the Russian Academy of Sciences, Krasnoyarsk, Russia.

**INRA** Institut National de Recherche Agronomique, Orléans, France.

### Molecular analyses

We DNA barcoded ten specimens of four Fabaceae-feeding *Phyllonorycter* species sampled in the Asian part of Russia: *P.ivani* (two larvae), *P.caraganella* (two adults and four larvae), *P.medicaginella* (Gerasimov, 1930) (one larva), and *P.viciae* (Kumata, 1963) (one larva) (Suppl. material 2: Table S2). In addition, 43 DNA barcodes, including 38 published sequences (De Prins et al. 2009; Laštůvka et al. 2013; Huemer and Hebert 2016; Mutanen et al. 2016), overall corresponding to 35 Fabaceae-feeding *Phyllonorycter* were added to the analysis (Suppl. material 2: Table S2).

DNA was extracted from larvae and adults using NucleoSpin® tissue XS kit, Macherey-Nagel, Germany according to the manufacturer’s protocol. The COI barcode fragment (658 bp) was amplified via PCR using the primers LCO (5’ GGT CAA CAA ATC ATA AAG ATA TTG G 3’) and HCO (5’ TAA ACT TCA GGG TGA CCA AAA AAT CA 3’) following standard conditions for the reaction (Folmer et al. 1994). Purification of PCR products was done using the NucleoSpin® Gel and PCR Clean-up kit Macherey-Nagel, Germany. For sequencing the Sanger method with Abi Prism® Big Dye®Terminator 3.1cycle sequencing kit was applied (25 cycles of 10s at 96 °C, 5s at 50 °C, 4 min at 60 °C). Sequencing was carried out using a 3500 ABI genetic analyzer. Sequence were revised and aligned in CodonCode Aligner 3.7.1. (CodonCode Corporation). DNA sequences, voucher data, images, and trace files were deposited in the Barcode of Life Data Systems (BOLD) (Ratnasingham and Hebert 2007; www.barcodinglife.org) and are available via public dataset: dx.doi.org/10.5883/DS-FABPHYL. The consensus sequences were also deposited in GenBank.

Barcode Index Numbers (BINs) were assigned by BOLD (Ratnasingham and Hebert 2013). Intra – and interspecific genetic distances were estimated using the Kimura 2-parameter and a multiparametric bootstrap test with 2000 iterations, with complete deletion (Kimura 1980). A Maximum Likelihood (ML) COI tree was built based on the Kimura 2-parameter model (Kimura 1980) and rooted using DNA barcode of the two *Sauterinahofmanniella* (Schleich, 1867) (Gracillariidae) specimens collected on *Lathyrus* sp. (Fabaceae) in Siberia. The outgroup was sequenced following the protocol described above. All computations were done in MEGA 7.0 (Kumar et al. 2016).

## Results

### Key to male genitalia and forewing pattern of the *haasi* species group and related species

**Table:** 

1	At least a part of markings margined with dark scales	**2**
–	Markings not margined with dark scales	**8**
2	White dorso-basal spot connected to basal streak	**3**
–	White dorso-basal spot not connected to basal streak or absent	**4**
3	White dorso-basal spot elongate towards base	*** telinella ***
–	White dorso-basal spot not elongate towards base	*** purgantella ***
4	First costal and dorsal strigulae connected at an obtuse angle	ivani ^*^
–	First costal and dorsal strigulae not connected and forming an acute angle	**5**
5	First and second costal strigulae connected or separated by a few black scales; left valva about 2× as wide as right one	*** scopariella ***
–	First and second costal strigulae well separated; left valva about 6× as wide as right one	**6**
6	Apex of first dorsal opposite first costal strigula	**7**
–	Apex of first dorsal opposite second costal strigula	tridentatae ^*^
7	Four costal strigulae	***haasi***
–	Five costal strigulae	***balansae***
8	Only two dorsal strigulae, the first forms a zig zag	*** deschkanus ***
–	More than two dorsal strigulae	**9**
9	First costal and dorsal strigulae forming a slightly angled fascia, if interrupted, the two strigulae are only slightly inclined	*** estrela ***
–	First costal and dorsal strigulae always separated and inclined at an acute angle	**10**
10	Subapical area without dark scales; saccus little differentiated from vinculum	*** baldensis ***
–	Subapical area with suffusion of dark scales; saccus filiform, well distinct from the vinculum	*** floridae ***

#### Phyllonorycter ivani sp. n.

Taxon classificationAnimaliaLepidopteraGracillariidae

http://zoobank.org/842E3172-2931-445C-98A4-F9603A68B250

**Figure 2.**
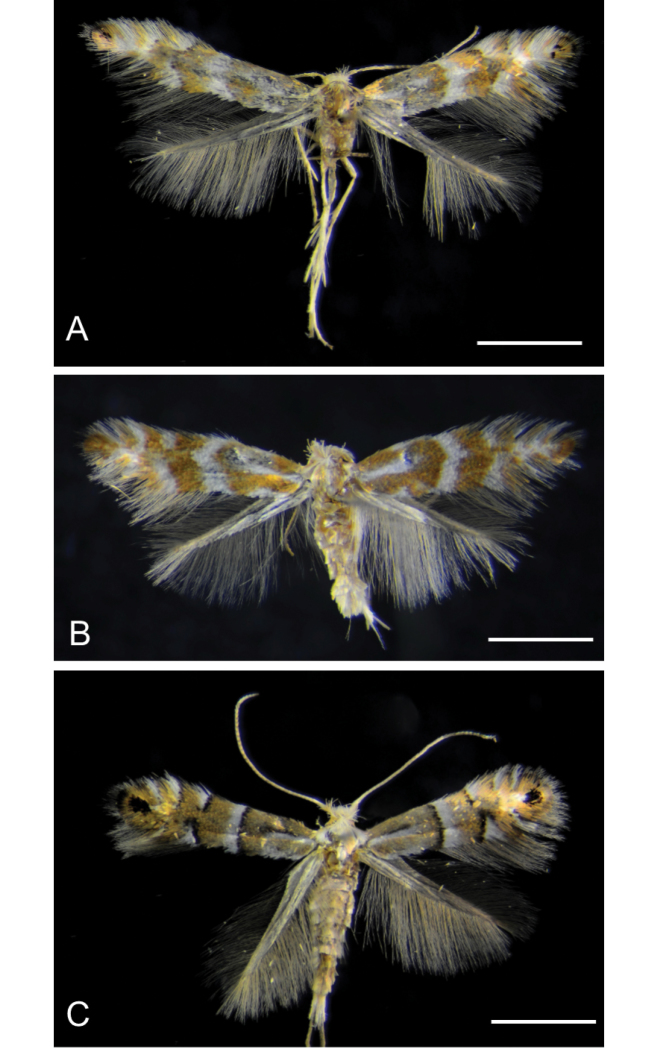
Adults of *Phyllonorycterivani* sp. n. and *P.caraganella***A, B***P.ivani* (holotype, ♂) , Russia, Krasnoyarsk, Akademgorodok, the river Yenisei, left bank, “Krasiviy bereg”, ex. *Caraganaarborescens*, 2.VII.2015, NK-69-15-6 (♂) , genitalia slide TRB4117♂ ; same location, date and host, NK-69-15-3 (♀), genitalia slide TRB4290♀ **C***P.caraganella*, Russia, Primorsky Krai, Rakovka, ex. *Caraganamanshurica*, 27.VII.2016, NK-184-16-8A (♀) , genitalia slide TRB4291♀. Scale bar: 1.2 mm.

**Figure 3.**
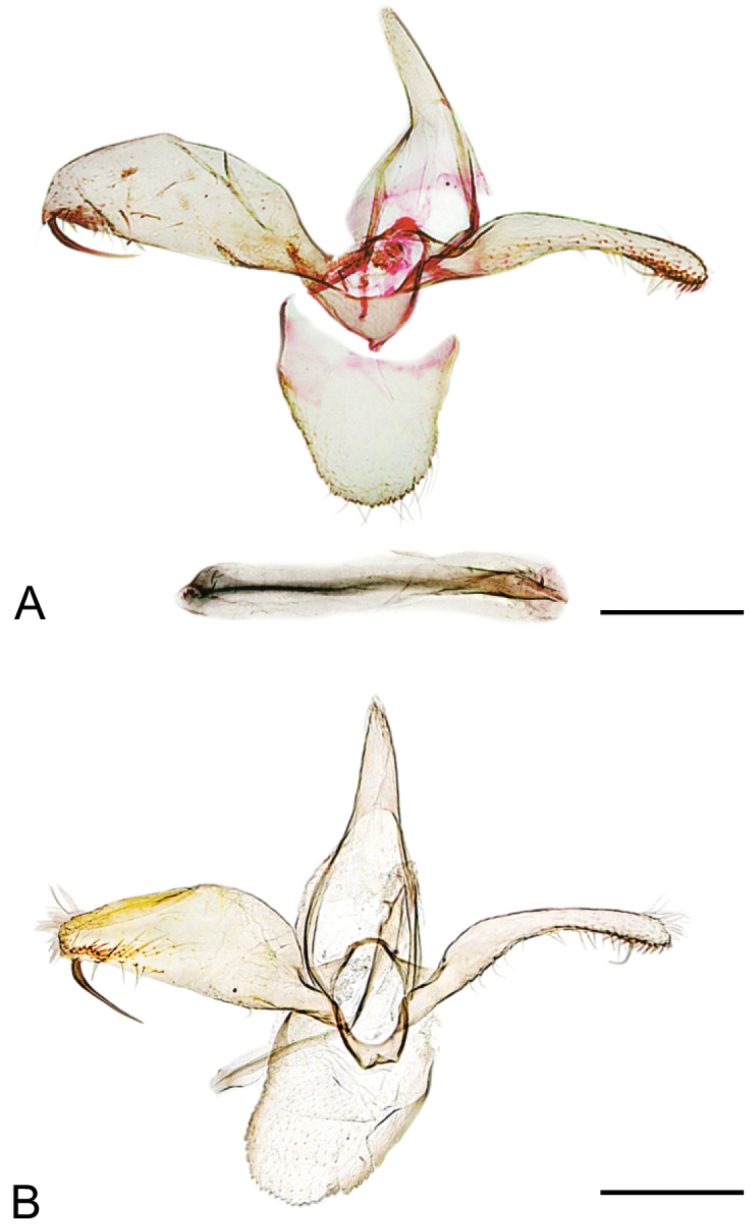
Male genitalia of *Phyllonorycterivani* sp. n. Russia, Krasnoyarsk, Akademgorodok, the river Yenisei, left bank, “Krasiviy bereg”, ex. *Caraganaarborescens*, 2.VII.2015 **A** holotype, NK-69-15-6 (♂) , genitalia slide TRB4117♂ , phallus removed **B** paratype, NK-69-15-1 (♂) , genitalia slide NK- 69- 15- 1♂ . Scale bar: 200 µm.

**Figure 4.**
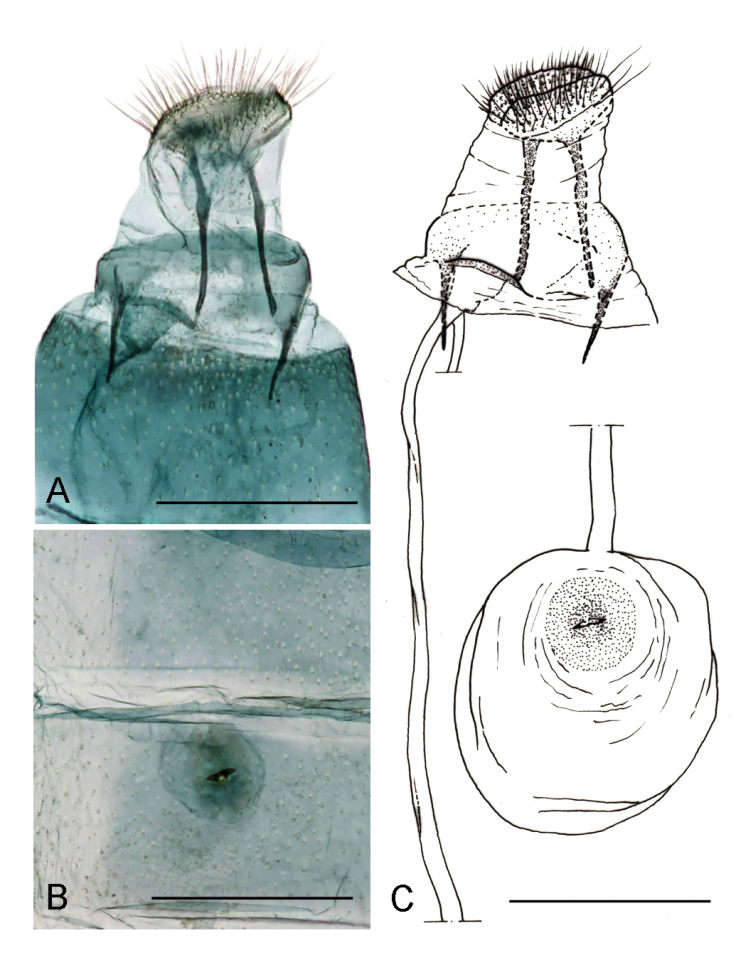
Female genitalia of *Phyllonorycterivani* sp. n. Russia, Krasnoyarsk, Akademgorodok, the river Yenisei, left bank, “Krasiviy bereg”, ex. *Caraganaarborescens*, 2.VII.2015, NK-69-15-3, genitalia slide TRB4290♀ **A** last segments of abdomen **B** signum **C** drawing of female genitalia based on the genitalia slide TRB4290♀ . Scale bar: 300 μm.

**Figure 5.**
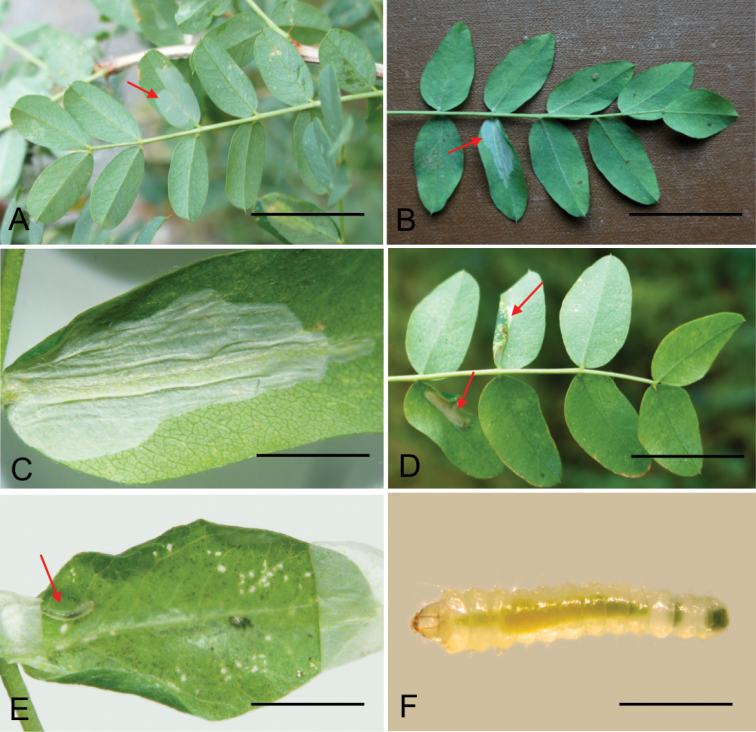
Biology of *Phyllonorycterivani* sp. n., *Caraganaarborescens*, Russia, Siberia **A** flat blotch mine (indicated by an arrow) on low side of the leaflet **B–C** tentiform mine with folded epidermis **D** the mine and leaf margin folded downward (an arrow) **E** opened mine with a larva (an arrow) inside **F** larva before pupation. **A** Transbaikal Krai, Chita, Viktory park, 11.VIII.2015 **B–F** Krasnoyarsk, the river Yenisei, left bank, “Krasiviy bereg”, 15.VIII.2014. Scale bars: 20 mm (**A–B, D, F**); 10 mm (**C**); 15 mm (**E**).

Figs 2A, B , 3 , 4 , 5

##### Diagnosis.

Forewing yellow ochre and white markings, with a basal streak, an angulated fascia in the median third and three costal and dorsal strigulae, all margined, often indistinctly, with darkish colour. Male genitalia asymmetric with a wide left valva, long spines apically and a thin right valva. Female genitalia with sterigma membranous and a large ostium bursae, signum consisting of an oval plate with two opposite spines in the centre.

The forewing pattern of *P.ivani* is similar to *P.caraganella* and *P.viciae*. It differs by the reduced or absent dark margins of all markings, a much angulated median fascia, an often present third strigula, and an indistinct apical spot, clearly defined in the other two species. In male genitalia, *P.ivani* is significantly different from *P.caraganella* by the asymmetrical valvae. For this character, *P.ivani* is similar to *P.viciae* but it is distinguishable for the just outlined saccus, which is very evident in *P.viciae*, a different curvature of the right valva and the sternum VIII rounded and not rectangular (Kumata 1963). In female genitalia, *P.ivani* differs from *P.caraganella* for the lobate posterior margin of the segment VII in the latter and for the spines in the signum which are opposite, on a horizontal plane, while are not aligned in *P.caraganella*. In *P.viciae* signum is similar to *P.ivani* but there is a very different fan-shaped lamella antevaginalis (Kumata 1963).

##### Type material.

Holotype ♂ (Fig. 2A): Russia, Krasnoyarsk Krai, Krasnoyarsk, Akademgorodok, the river Yenisei (left bank), “Krasiviy bereg”, 55.99N, 92.76E, 256 m, ex. *Caraganaarborescens*, 2.VII.2015 (larva), 8.VII.2015 em., N Kirichenko leg., NK-69-15-6, genitalia slide TRB4117♂ (SIF SB RAS).

##### Paratypes.

6♂, 2♀ (Fig. 2B). Same location, date and host plant, N Kirichenko leg., NK-69-15-3 (♀), genitalia slide TRB4290♀ (MSNV); NK-69-15-9 (♂), genitalia slide TRB4129♂ (MSNV); NK-69-15-8 (♂), genitalia slide TRB4128♂ (MSNV); NK-69-15-1 (♂), genitalia slide NK-69-15-1♂; NK-69-15-2 (♂), genitalia slide NK-69-15-2♂; NK-95-15-4 (♂), NK-95-15-5 (♂); NK-95-15-7 (♂) (SIF SB RAS).

##### Further material examined.

2 larvae. 1 larva, Russia, Transbaikal (Zabaikalsky) Krai, Chita, Viktory park, 52.03N, 113.50E, 75 m, 11.VIII.2015, *C.arborescens*, N Kirichenko leg., field ID: NK-261-15, sample ID NK510, process ID: MICRU065-15; 1 larva, Krasnoyarsk Krai, Krasnoyarsk, Akademgorodok, Yenisei river bank, “Krasiviy bereg”, 55.99N, 92.76E, 256 m, 15.VIII.2014, N Kirichenko leg., *C.arborescens*, filed ID: Kr-22, sample ID NK333, process ID: ISSIK282-14 (INRA).

##### Etymology.

The species name, *ivani* is derived from the first name of Natalia Kirichenko’s father, Ivan, who has continuously supported her interest in entomology.

##### Description.

Male and female. Alar expanse: 6.5–7 mm (Fig. 2A, B).

*Head.* Vertex rough, white, with mixture of ochreous piliform scales anteriorly; frons smooth, with broad, lustrous white scales. Antenna light ochre, length approximately 0.7× that of forewing, each flagellomere ringed with dark brown apically, scape and pedicel yellow white, the first sometimes spotted with dark brown above, pecten of a few piliform scales. Maxillary and labial palpi white, the first very reduced, about 1/5 of the labial palpi.

*Thorax* (Fig. 2A–B). Yellow ochre with three longitudinal white lines, venter white. Forewing yellow ochre to orange, with a basal streak at basal one third, an angulated fascia in the median third and three costal and dorsal white strigulae, all the signs are slightly margined with dark colour, sometimes third dorsal strigula not perceptible; an indistinct apical dark spot, almost always represented by a few dark scales; cilia whitish. Hindwing pale grey, cilia pale ochreous grey. Legs mostly fuscous dorsally, white ventrally, fore and mid tarsi more or less annulated with brownish, hind tarsi white.

*Abdomen.* Sternum VIII of male shorter than right valve, with a round apex.

*Male genitalia* (Fig. 3A–B). Tegumen long, pointed, no apical microsetae. Valvae asymmetrical: left valva broad, variable in width, much broader near middle, about three times the width of right valva, and with a stout, sinuate spine arising near apex, length of spine about the width of valva; right valva slender, curved, with a big seta subapically. Vinculum short, saccus triangular but just outlined. Phallus slender, with a small subapical spine (Fig. 3A), length approximately equal to right valva.

*Female genitalia* (Fig. 4A–C). Papillae anales rather reduced, posterior apophyses almost twice the length of the anterior one (Fig. 4A, C). Sterigma membranous, ostium bursae rather large, antrum narrower, approximately half of the ostium, weakly sclerotized. Ductus bursae thin, membranous, extended to the segment II; bursa rounded with signum consisting of two opposite spines, arranged horizontally, in the centre of a small sclerotized plate (Fig. 4B, C). Ductus spermathecae with efferent canal forming 35–36 coils of equal diameter.

##### Biology.

(Fig. 5). The mine is similar to other *Phyllonorycter* species. The early mine is a whitish flat blotch on the lower side of the leaflet (Fig. 5A). The long epidermal tunnel preceding the blotch mine, as often present in the mines of *Phyllonoryctercaraganella*, has not been observed in *P.ivani* mines. The mine usually begins near the base of the leaflet, growing towards the leaflet tip or in the middle of the leaflet. Later it becomes a tentiform blotch with 2–4 folds on the lower epidermis covering the mine (Fig. 5B, C). Silken threads, which the larva attaches on the lower epidermis inside the mine, contract the epidermis, pulling the leaflet margins downward (Fig. 5D). The resulting narrowed leaflet may help to find the mine when examining leaves from the upper side. The mine may occupy the entire leaflet (Fig. 5D). Frass is in loose gains or in small batches, covered by silk. The larva primarily consumes spongy parenchyma and in the late stage it fragmentally gnaws the layer of palisade parenchyma. The latter results in the presence of small transparent dots that could be observed from the upper side of the leaf. Larva greenish white, before pupation greenish yellow (Fig. 5D, E). Pupation in the mine.

##### Phenology.

In Siberia, in 2014–2015, mines with late instar larvae were found in early July and with young larvae in August suggesting that the insect develops in two generations. The first generation (egg laying) likely starts in late May – beginning of June and lasts till middle of July (adult appearance), the second starts in mid-July and lasts till the end of August – early September. The overwintering stage of this species is unknown.

##### Ecology and host plant range.

The host plant is *Caraganaarborescens* (Fabaceae). So far, *P.ivani* has been found in suburban areas. Indeed, the type locality is on the outskirts of Krasnoyarsk (Krasnoyarsk Krai, Russia) where the bushes of its host plant are planted as an ornamental fence along the promenade on the left river bank of the river Yenisei. In Chita (Transbaikal Krai), the mines were found on bushes of *C.arborescens* in the city park.

##### Distribution.

Russia: Central Siberia (Krasnoyarsk Krai, Krasnoyarsk), Eastern Siberia (Transbaikal Krai, Chita). In 2014–2017, no mines of *P.ivani* were found on *Caragana* spp. in other regions of Siberia: Tyumen, Omsk, Novosibirsk Oblasts, Khanty-Mansi Autonomous Okrug, Tomsk, Kemerovo, Irkutsk Oblasts, Altai Krai, the Republics of Tuva and Buryatia, neither in the Russian Far East (Amur Oblast, Sakhalin Island). However, it is highly likely that the species occurs in Eastern Siberia, on the territory between Krasnoyarsk and Transbaikal Krais.

#### Phyllonorycter caraganella

Taxon classificationAnimaliaLepidopteraGracillariidae

(Ermolaev, 1986)

**Figure 6.**
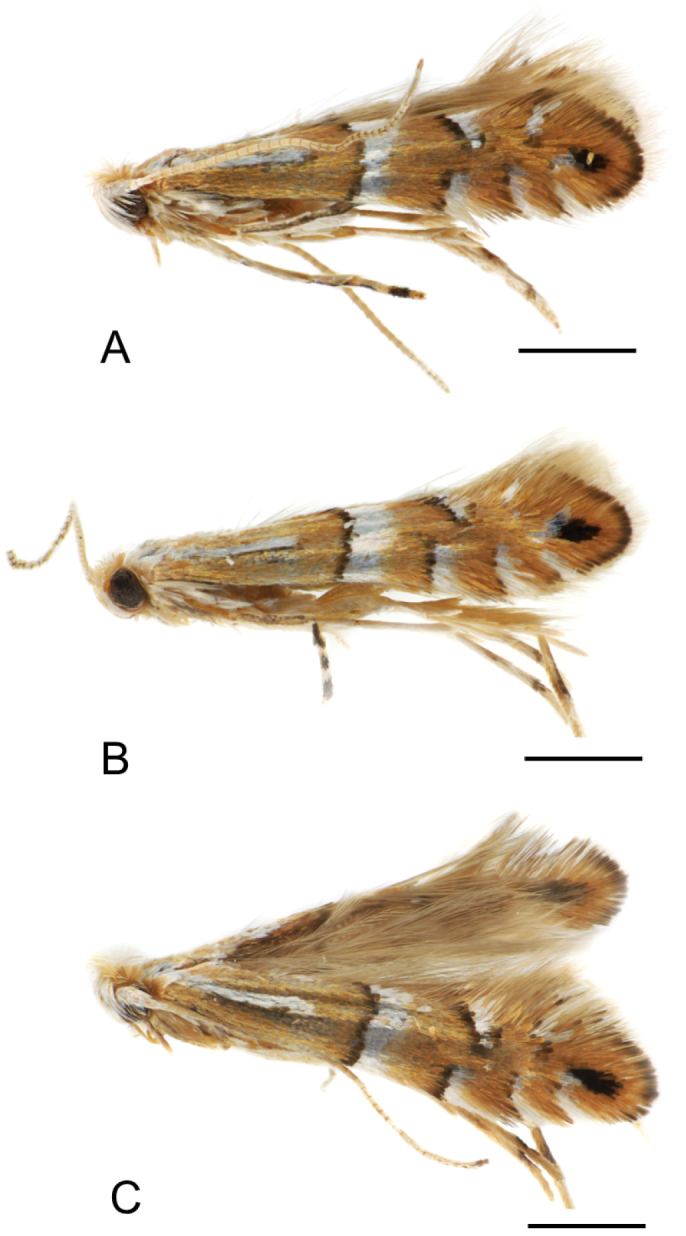
Male adults of *Phyllonoryctercaraganella*. Russia, Primorsky Krai, 27.VII.2016, ex. *Caraganamanshurica*, N. Kirichenko col. Sampling location, field ID **A** Glukhovka, NK-148-16-13A **B–C** Rakovka, NK-184-16-9A, NK-184-16-12A. Scale bar: 0.5 mm.

**Figure 7.**
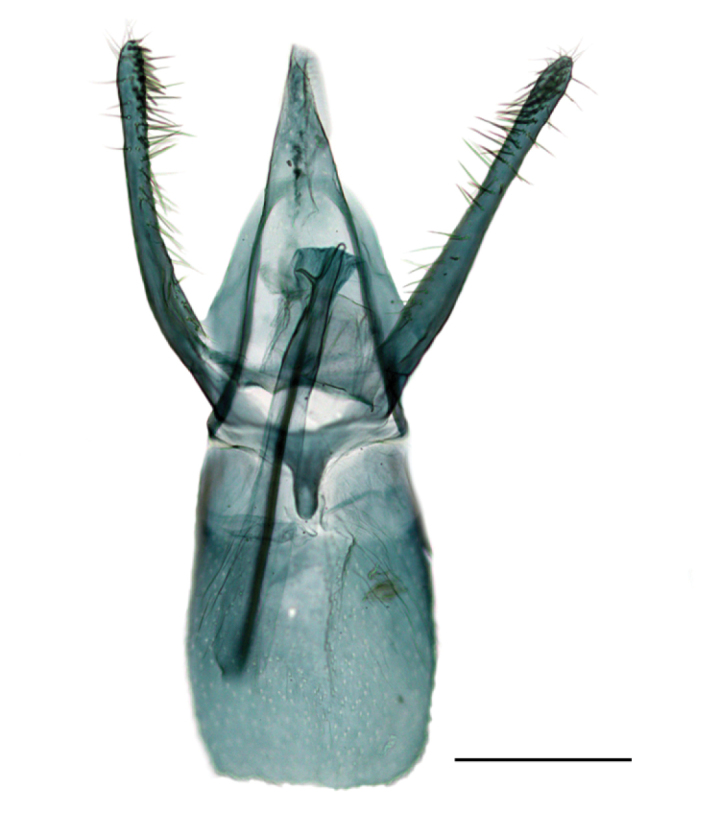
Male genitalia of *Phyllonoryctercaraganella*. Russia, Primorsky Krai, Rakovka, ex. *Caraganamanshurica*, N° 137, NK-184-16-11° (♂) , genitalia slide TRB4292♂. Scale bar: 250 µm.

**Figure 8.**
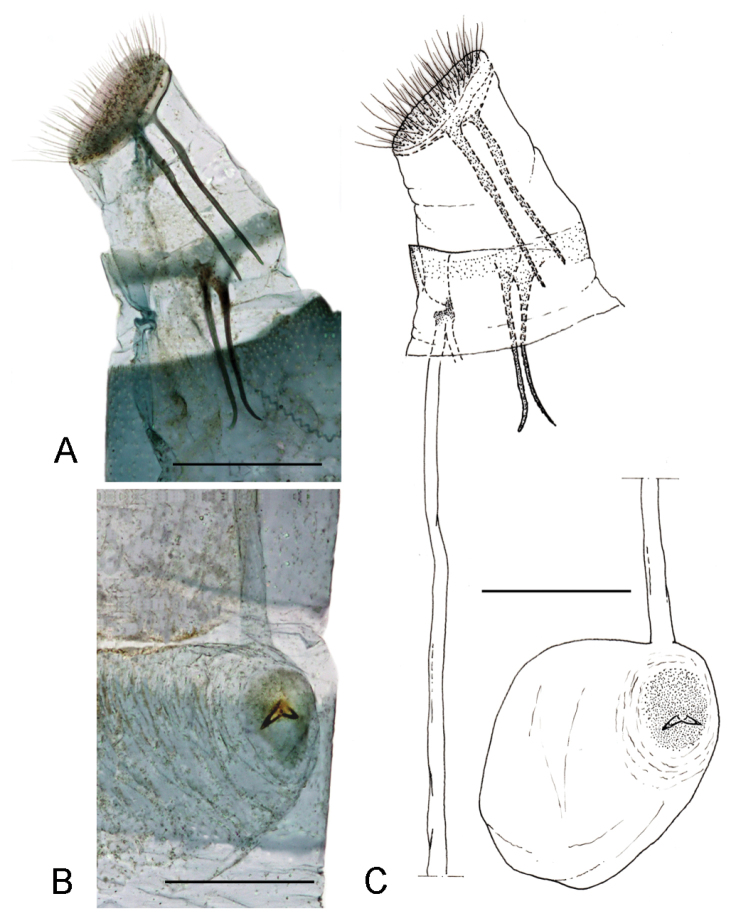
Female genitalia of *Phyllonoryctercaraganella*. Russia, Primorsky Krai, Rakovka, ex *Caraganamanshurica*, 27.VII.2016, N° 137, NK 184-16-3A, genitalia slide TRB4295♀ **A** last segments of abdomen **B** signum **C** drawing of female genitalia based on the genitalia slide TRB4295♀. Scale bar: 300 μm.

**Figure 9.**
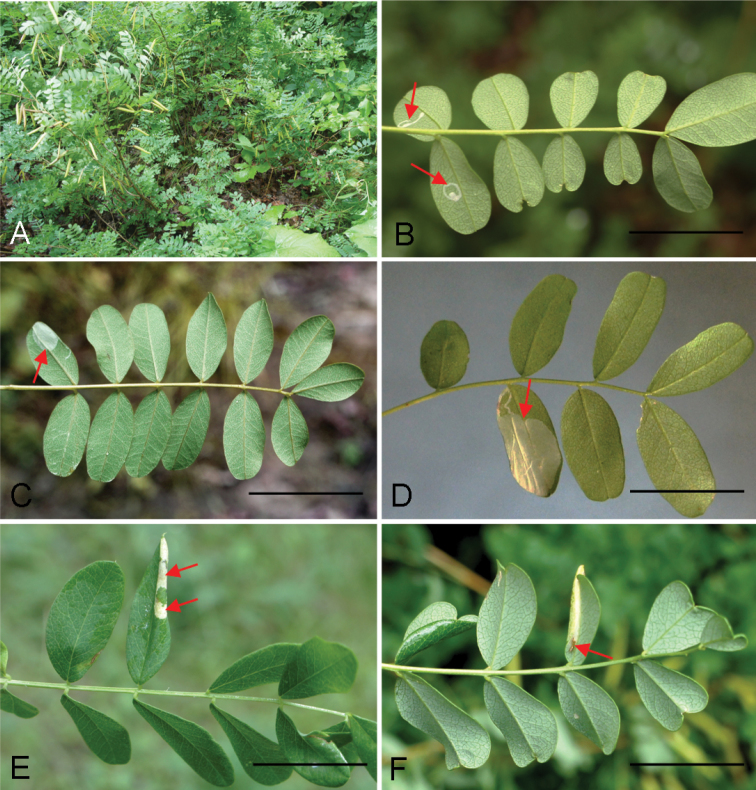
Biology of *Phyllonoryctercaraganella* (Ermolaev, 1986) on *Caraganamanshurica*, Russian Far East, Primorsky Krai, 27.VII.2016 **A** sampling plot **B** young epidermal tunnel mines on low side of the leaflet (indicated by the arrows) **C–D** flat blotch mine with the preceding epidermal tunnel (arrow) **E** tentiform mine with leaf margin folded downwards and with whitish “windows” – the regions of eaten out palisade parenchyma in the mine (arrows) **F** same mine (see **E**) from lower side of the leaflet, with pupal exuvium protruding the mine (arrow). Scale bar: 20 mm.

Figs 2C , 6 , 7 , 8 , 9

##### Diagnosis.

Forewing bright yellow ochre, with a basal streak, a not angulated fascia in the median third and three costal and two dorsal strigulae, all markings clearly margined with dark colour. Male genitalia symmetrical with long thin valvae. Female genitalia with a rounded margin of sternum VII, signum consisting of an oval plate with two opposite spines not aligned horizontally.

Because of the symmetrical male genitalia, *P.caraganella* is close to *P.fabaceaella* (Kuznetzov, 1978) and *P.kuznetzovi* Ermolaev (Suppl. material 1: Table S1) but differs from the first by the presence of a large saccus (Kuznetzov 1981) and from the second by a very different shape of phallus and sternum VIII (Ermolaev 1982).

##### Material examined.

6♂, 2♀ 4 larvae (Figs 2C, 6). 1♂, Russia, Primorsky Krai, Glukhovka, vodorazdel, 43.74N, 132.13E, 68 m, ex. *Caraganamanshurica*, 27.VII.2016, N Kirichenko leg., field ID NK-148-16-13A (♂) (SIF SB RAS); 2♂, 1♀, Primorsky Krai, Rakovka, forested area, 43.80N, 132.19E, 140 m, ex. *C.manshurica*, 27.VII.2016, N Kirichenko leg., NK-184-16-8A (♀) (MSNV), NK-184-16-9A (♂), NK-184-16-12A (♂), genitalia slide NK-184-16-12A♂ (SIF SB RAS); 1♂, 1♀, Primorskiy Krai, Rakovka, ex *Caraganamanshurica*, 27.VII.2016, N Kirichenko leg., N° 137, NK-184-16-11° (♂), genitalia slide TRB4292♂, NK-184-16-3° (♀), genitalia slide TRB4295♀ (MSNV); 2♂, Primorsky Krai, Rakovka, forested area, 43.80N, 132.19E, 140 m, ex. *C.manshurica*, 27.VII.2016, N Kirichenko leg., NK-184-16-1A (♂) (sample ID NK526, process ID SIBLE015-17), NK-184-16-2A (♂) (NK527, SIBLE016-17); 2 larvae, same place, date and host plant, N Kirichenko leg., NK-184-16-1 (NK522, SIBLE011-17), NK-184-16-2 (NK523, SIBLE012-17); 2 larvae, Primorsky Krai, Glukhovka, vodorazdel, 43.74N, 132.13E, 68 m, *C.manshurica*, 27.VII.2016, N Kirichenko leg., NK-185-16-1 (NK524, SIBLE013-17), NK-185-16-2 (NK525, SIBLE014-17) (INRA).

##### Description.

Male and female. Alar expanse: 6.5–7.2 mm (Figs 2A, B, 7, 8).

*Head.* As in the previous species, dark scales on scape are not present.

*Thorax* (Fig. 2A–B). Yellow ochre with three longitudinal white lines, venter white. Forewing yellow ochre, with a basal streak at basal one third, a fascia in the median third (straight or weakly angled) and three costal and two dorsal white strigulae, all the signs are clearly margined with dark brown; a subapical elliptical dark spot; cilia whitish. Hindwing pale grey, cilia pale ochreous grey. Legs mostly fuscous dorsally, white ventrally, fore and mid tarsi more or less annulated with brownish, hind tarsi white.

*Abdomen.* Sternum VIII rectangular, shorter than valva.

*Male genitalia* (Fig. 7). Tegumen long, pointed, no apical microsetae. Valvae symmetrical, thin, parallel-sided, slightly curved. Vinculum short, saccus pronounced, with a round apex. Phallus slender, with a small subapical spine, slightly longer than valva.

*Female genitalia* (Fig. 8A–C) Papillae anales rather reduced, posterior apophyses slightly longer than the anterior one (Fig. 8A, C). Sterigma membranous; with a rounded margin of sternum VII; a rather large ostium bursae, antrum membranous, strongly folded in the conjunction with ductus. Ductus bursae thin, membranous, extended to the segment II. Bursa rounded with signum consisting of two opposite spines, not aligned horizontally, in the centre of a small sclerotized plate (Fig. 8B, C). Ductus spermathecae with efferent canal forming 30 coils of equal diameter.

##### Biology.

(Fig. 9). The mine is a whitish blotch on the leaflet underside. In contrast to *P.ivani*, the mine of *P.caraganella* often starts with a relatively long narrow, hardly widening, epidermal tunnel, that proceeds into a flat blotch mine (Fig. 9B–D). The later mine is tentiform, with leaf margins contracted downwards, reminding of the mine of *P.ivani* (Fig. 8E). Tentiform blotch with 2–5 folds, most often occupying the whole leaflet (Fig. 9D). The larva primarily consumes the spongy parenchyma and later feeds on palisade parenchyma, gnawing rather large “windows” visible from the upper side of the leaflet (Fig. 9E). Pupation occurs in the mine. After adult emergence, pupal exuviae can be found in the corner of the mine close to leaflet base (Fig. 9F).

##### Phenology.

Two generations. In Russian Far East, vacated tentiform mines of the first generation and young mines (epidermial tunnels) of the second generation were found in the end of July 2016. It is unknown how the species hibernates.

##### Ecology and host plant range.

(Fig. 9A). The host plant is *Caraganamanshurica* (syn. *C.fruticosa* (Pallas) Besser) (Fabaceae). This species is very similar to *C.arborescens* (Koropachinsky and Vstovskaya 2012). The bushes of *C.manshurica* with *P.caraganella* mines were found in the canopy in the broadleaf forest in the southern part of Primorsky Krai.

##### Distribution.

Russia, Russian Far East: Khasansky District (Barabash) (Ermolaev 1986), Ussurijsk District – Komarov Mountain-Taiga Station, village Gornotayezhnoe (SV Baryshnikova: personal communication), around the villages Glukhovka and Rakovka (present paper). In 2017, no mines of *P.caraganella* were found on *Caragana* spp. in the southern part of the Island Sakhalin (Russian Far East), nor could we find *P.caraganella* in Siberia during our extensive surveys in 2015–2017.

##### Remarks.

The holotype (♂) and paratypes (4♂ and 7♀) that, according to Ermolaev (1986), are being stored in the Zoological Institute, Russian Academy of Science (Saint Petersburg, Russia), are not located there (SV Baryshnikova: personal communication). For a note about VP Ermolaev’s research journey and the destiny of his gracillariid collections see Kirichenko et al. (2018b).

## Molecular data

**Figure 10.**
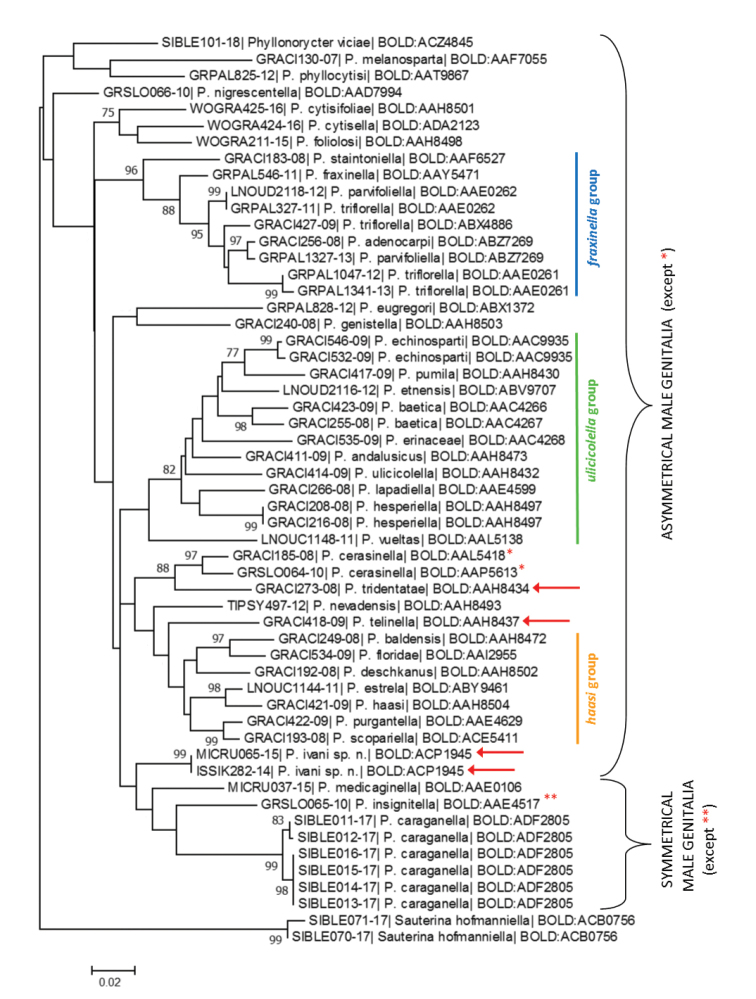
A Maximum Likelihood COI tree of the Fabaceae-feeding *Phyllonorycter* generated with the K2P nucleotide substitution model. Each specimen is identified by its Process ID code (see Table S2) and Barcode Index Number (BIN). Branch lengths are proportional to the number of substitutions per site. The percentage of trees in which the associated taxa clustered together is shown next to the branches, with the bootstrap values >70. Species indicated by red arrow highly similar to *haasi* group morphologically (male genitalia), but not genetically. *P.cerasinella has symmetrical male genitalia, **P.insignitella asymmetrical (Laštůvka and Laštůvka 2006).

We obtained barcode data for 53 Fabaceae-feeding *Phyllonorycter* specimens belonging to 44 BINs and 39 species (Suppl. material 2: Table S2, Fig. 10). DNA barcodes of four *Phyllonorycter* species were assigned to more than one BIN in BOLD, i.e., *P.baetica* Laštůvka & Laštůvka, 2006, *P.cerasinella* (Reutti, 1853) and *P.parvifoliella* (Ragonot, 1875) were assigned to two BINs each and *P.triflorella* (de Peyerimhoff, 1871) to three BINs (Fig. 10). DNA barcodes of *P.ivani* and *P.caraganella* were novel to BOLD and were assigned their own unique BINs, BOLD:ACP1945 and BOLD:ADF2805, respectively (Fig. 10). On the Maximum Likelihood COI tree, species with asymmetrical male genitalia grouped together in three main, weakly supported, species groups: the *fraxinella*, *ulicicolella* and *haasi* groups. Some species with asymmetrical male genitalia that did not fit any of the three groups based on their morphology (Laštůvka and Laštůvka 2006) occupied a relatively isolated position on the COI tree (Fig. 10). Species with symmetrical male genitalia (*P.medicaginella* and *P.caraganella*) clustered together, but including a species with asymmetrical male genitalia, *P.insignitella* (Zeller) (Fig. 10).

The nearest neighbours of the new species *P.ivani* were both *P.purgantella* (Chrétien, 1910) and *P.scopariella* (Zeller, 1846) from the *haasi* group (asymmetrical male genitalia) with 6.3% divergence, followed by *P.medicaginella* (symmetrical male genitalia clade) with 6.4% divergence (Table 1). The new species *P.ivani* did not fall within the *haasi* species group but it clustered next to it (Fig. 10), which agrees with the features of its asymmetrical male genitalia (Fig. 3). *Phyllonoryctertelinella* and *P.tridentatae* that, as *P.ivani*, are morphologically highly similar to the *haasi* group, did not enter the *haasi* clade on the COI tree (Fig. 10). The minimum interspecific genetic distance between the two *Caragana*-feeding species *P.ivani* and *P.caraganella* was 8.6% (Fig. 10; Table 1). No evidence for mitochondrial introgression between these two species was detected. The intraspecific distance in *P.caraganella* (based on DNA barcodes of six individuals), varied from 0 to 1.1%. No genetic divergence was found between the two specimens of *P.ivani* collected from the two distant localities in Siberia (Krasnoyarsk in Central Siberia vs. Chita in Eastern Siberia).

**Table 1.** Intra- and interspecific genetic divergences in DNA barcode fragments (COI mtDNA) between *Phyllonorycterivani* sp. n. and the close neighbours – *Phyllonorycter* spp. with the asymmetrical male genitalia from *haasi* group (see Nr. 2, 3, 5, 7-12, 14) and *Phyllonorycter* spp. with the symmetrical male genitalia (4, 6, 13)*.

№	**Species**	***P.ivani* sp. n.**	*** P. purgantella ***	*** P. scopariella ***	*** P. medicaginella ***	*** P. nevadensis ***	*** P. insignitella ***	*** P. estrela ***	*** P. deschkanus ***	*** P. haasi ***	*** P. telinella ***	*** P. floridae ***	*** P. baldensis ***	*** P. caraganella ***	*** P. tridentatae ***
		1	2	3	4	5	6	7	8	9	10	11	12	13	14
1	*P.ivani* sp. n.	[**0**]													
2	*P.purgantella* (Chrétien, 1910)	6.3	[−]												
3	*P.scopariella* (Zeller, 1846)	6.3	1.5	[−]											
4	*P.medicaginella* (Gerasimov, 1930)	6.4	7.4	7.4	[−]										
5	*P.nevadensis* (Walsingham, 1908)	6.5	6.0	5.9	7.4	[−]									
6	*P.insignitella* (Zeller, 1846)	7.1	8.9	8.8	7.7	9.3	[−]								
7	*P.estrela* Laštůvka & Laštůvka, 2006	7.2	4.3	4.4	8.7	6.5	9.6	[**3.1**]							
8	*P.deschkanus* Laštůvka & Laštůvka, 2006	7.7	5.2	4.7	8.0	7.2	8.6	4.7	[−]						
9	*P.haasi* (Rebel, 1901)	7.9	5.4	5.6	9.1	6.7	9.7	2.3	5.6	[−]					
10	*P.telinella* Laštůvka & Laštůvka, 2006	8.0	6.5	6.5	8.2	6.9	10.2	6.0	6.4	6.7	[−]				
11	*P.floridae* Laštůvka & Laštůvka, 2006	8.2	5.7	5.7	8.4	7.4	9.4	5.4	5.1	6.3	7.0	[−]			
12	*P.baldensis* Laštůvka & Laštůvka, 2006	8.2	5.2	5.6	8.7	7.7	10.0	6.1	5.6	7.0	7.7	3.5	[−]		
13	*P.caraganella* (Ermolaev, 1986)	8.6	8.6	8.5	8.7	9.1	8.8	10.2	8.7	10.4	9.2	8.5	9.5	[**0−1**]	
14	*P.tridentatae* Laštůvka & Laštůvka, 2006	8.7	7.9	8.1	8.9	8.9	10.0	8.6	8.1	10.0	8.4	8.2	9.3	10.1	[−]

*Kimura 2-parameter (K2P) distances (%); minimal pairwise distances are given for each species pair; values in square brackets represent maximal intraspecific distances. [−] no data because a single specimen was DNA-barcoded.

## Discussion

The new species, *P.ivani* is the second *Phyllonorycter* species described from *Caragana* (Fabaceae). This species is clearly distinguishable from the other *Caragana*-feeding species, *P.caraganella* by external morphology (forewing pattern) and highly different male genitalia, i.e., asymmetric in *P.ivani* and symmetrical in *P.caraganella*. High genetic divergence found between these two gracillariid species, suggests that the plant genus *Caragana* has been colonized at least twice independently in the Eastern Palearctic.

Despite extensive field surveys in the Asian part of Russia, the new species has so far been detected only in two locations in Central and Eastern Siberia, whereas *P.caraganella* has been found exclusively in the southern territory of the Russian Far East. Both species are monophagous, feeding exclusively on *C.arborescens* (*P.ivani*) and *C.manshurica* (*P.caraganella*) respectively. The natural ranges of these plants do not overlap: *C.arborescens* occurs in the forest and forest-steppe zones in Siberia, China, Mongolia, and Kazakhstan, whereas *C.manshurica* grows in the Russian Far East, northeast China, and Korea (Liu et al. 2018). *Caraganaarborescens* and *C.manshurica* are very similar morphologically (Koropachinsky and Vstovskaya 2012). *Caraganaarborescens* was introduced to the European part of Russia, some European countries and North America for ornamental reasons and to protect landscapes (hedging, screening or wind-breaking). In North America, it became naturalized and weedy (Shortt and Vamosi 2012). However, no records of *Phyllonorycter* species on *Caragana* are known yet from the neocolonized range of *C.arborescens* (De Prins and De Prins, 2018).

The genus *Caragana* has 96 described species (The Plant List 2018). Bearing in mind the fact that *P.ivani* and *P.caraganella* feed on different host plants, it is likely that other new *Phyllonorycter* species will be found feeding on other *Caragana* species. More fieldwork and rearing efforts are needed to test this hypothesis.

DNA barcode data weakly support the different Fabaceae-feeding species groups, but data on more loci are needed to infer the phylogenetic interrelationships of those groups and the evolution of asymmetric genitalia (Doorenweerd 2016).

By its asymmetric male genitalia and specific valval structures, *P.ivani* is similar to the *haasi* group. According to their DNA barcodes, two species of the *haasi* group, *P.purgantella* and *P.scopariella* are the nearest neighbours of *P.ivani* (Fig. 10). The identification of species within the *haasi* group is very difficult, due to the lack of diagnostic morphological characters. Indeed, species belonging to this group show a very uniform wing pattern and minor morphological characters in male genitalia, such as a subapical bristle or a small basal tuft of setae on the right valva (Laštůvka and Laštůvka 2006). Female genitalia in this species group are also poorly differentiated. A further source of confusion is that two other species groups of *Phyllonorycter* show a similar structure of the male genitalia. The first, the *hilarella* group, feeds on Salicaceae (Davis and Deschka 2001) and the second, the *acerifoliella* group, feeds on Sapindaceae (Gregor and Povolný 1950). Genitalia of both sexes and, very often, the forewing pattern of these two species groups are very similar to those of the Fabaceae groups. More DNA sequence data are needed to test the validity of those species groups and their phylogenetic relationships.

## Supplementary Material

XML Treatment for Phyllonorycter ivani

XML Treatment for Phyllonorycter caraganella

## Supplementary materials

Supplementary material 1 Table S1 Explanation note: Fabaceae-feeding *Phyllonorycter* of the world. This dataset is made available under the Open Database License (http://opendatacommons.org/licenses/odbl/1.0/). The Open Database License (ODbL) is a license agreement intended to allow users to freely share, modify, and use this Dataset while maintaining this same freedom for others, provided that the original source and author(s) are credited.Natalia Kirichenko, Paolo Triberti, Carlos Lopez-Vaamonde

Supplementary material 2 Table S2 Explanation note: Fabaceae-feeding *Phyllonorycter* species involved in the study. Where relevent, genitalia preparation number is given in square brackets in the Life stage column. Both the Process ID and Sample ID codes link the record in the BOLD database and the voucher specimen from which the sequence is derived. This dataset is made available under the Open Database License (http://opendatacommons.org/licenses/odbl/1.0/). The Open Database License (ODbL) is a license agreement intended to allow users to freely share, modify, and use this Dataset while maintaining this same freedom for others, provided that the original source and author(s) are credited.Natalia Kirichenko, Paolo Triberti, Carlos Lopez-Vaamonde
